# Immune changes in pregnancy: associations with pre-existing conditions and obstetrical complications at the 20th gestational week—a prospective cohort study

**DOI:** 10.1186/s12916-024-03797-y

**Published:** 2024-12-18

**Authors:** David Westergaard, Agnete Troen Lundgaard, Kilian Vomstein, Line Fich, Kathrine Vauvert Römmelmayer Hviid, Pia Egerup, Ann-Marie Hellerung Christiansen, Josefine Reinhardt Nielsen, Johanna Lindman, Peter Christoffer Holm, Tanja Schlaikjær Hartwig, Finn Stener Jørgensen, Anne Zedeler, Astrid Marie Kolte, Henrik Westh, Henrik Løvendahl Jørgensen, Nina la Cour Freiesleben, Karina Banasik, Søren Brunak, Henriette Svarre Nielsen

**Affiliations:** 1 Novo Nordisk Foundation Center for Protein Research, Faculty of Health and Medical Sciences, University of Copenhagen, Copenhagen, Denmark; 2 Methods and Analysis, Statistics Denmark, Copenhagen, Denmark; 3 Department of Obstetrics and Gynecology, Copenhagen University Hospital Hvidovre, Hvidovre, Denmark; 4 Department of Clinical Microbiology, Copenhagen University Hospital Hvidovre, Hvidovre, Denmark; 5 Department of Clinical Biochemistry, Copenhagen University Hospital Hvidovre, Hvidovre, Denmark; 6 Department of Clinical Medicine, Faculty of Health and Medical Sciences, University of Copenhagen, Copenhagen, Denmark

**Keywords:** Women’s health, COVID-19, Immunology, Pregnancy, Risk prediction, Pre-eclampsia, Gestational diabetes

## Abstract

**Background:**

Pregnancy is a complex biological process and serious complications can arise when the delicate balance between the maternal and semi-allogeneic fetal immune systems is disrupted or challenged. Gestational diabetes mellitus (GDM), pre-eclampsia, preterm birth, and low birth weight pose serious threats to maternal and fetal health. Identification of early biomarkers through an in-depth understanding of molecular mechanisms is critical for early intervention.

**Methods:**

We analyzed the associations between 47 proteins involved in inflammation, chemotaxis, angiogenesis, and immune system regulation, maternal and neonatal health outcomes, and the baseline characteristics and pre-existing conditions of the mother in a prospective cohort of 1049 pregnant women around the 20th gestational week. We used Bayesian linear regression models to examine the impact of risk factors on biomarker levels and Bayesian cause-specific parametric proportional hazards models to analyze the effect of biomarkers on maternal and neonatal outcomes. We evaluated the predictive value of baseline characteristics and 47 proteins using machine-learning models and identified the most predictive biomarkers using Shapley additive explanation scores.

**Results:**

Associations were identified between specific inflammatory markers and several conditions, including maternal age and pre-pregnancy body mass index, chronic diseases, complications from prior pregnancies, and COVID-19 exposure. Smoking during pregnancy affected GM-CSF and 9 other biomarkers. Distinct biomarker patterns were observed for different ethnicities. Within obstetric complications, IL-6 inversely correlated with pre-eclampsia risk, while birth weight to gestational age ratio was linked to markers including VEGF and PlGF. GDM was associated with IL-1RA, IL-17D, and eotaxin-3. Severe postpartum hemorrhage correlated with CRP, IL-13, and proteins of the IL-17 family. Predictive modeling yielded area under the receiver operating characteristic curve values of 0.708 and 0.672 for GDM and pre-eclampsia, respectively. Significant predictive biomarkers for GDM included IL-1RA and eotaxin-3, while pre-eclampsia prediction yielded the highest predictions when including MIP-1β, IL-1RA, and IL-12p70.

**Conclusions:**

Our study provides novel insights into the interplay between preexisting conditions and immune dysregulation in pregnancy. These findings contribute to our understanding of the pathophysiology of obstetric complications and the identification of novel biomarkers for early intervention(s) to improve maternal and fetal health.

**Supplementary Information:**

The online version contains supplementary material available at 10.1186/s12916-024-03797-y.

## Background

During pregnancy, there is a complex interaction between the maternal immune system and the semi-allogeneic fetus [1]. The maternal immune system is highly regulated throughout pregnancy, and successful pregnancy requires a balance between tolerance and suppression. Obstetric complications are common, affecting more than one in four pregnancies, and immune dysregulation is involved in the pathogenesis of a range of complications, including gestational diabetes mellitus (GDM), pre-eclampsia, preterm birth, and low birth weight [2, 3]. Immune dysregulation may be due to preexisting or subclinical diseases, complications from prior pregnancies, or viral infections. Indeed, during pregnancy, the maternal immune system seems to be more challenged by viral infections such as influenza [4, 5], respiratory syncytial virus (RSV) [6], severe acute respiratory syndrome (SARS-CoV) [7, 8], and Middle East respiratory syndrome (MERS-CoV) [9]. Nonetheless, many pregnancy complications arise without prior known risk factors. Consequently, identifying biomarkers that are present early in pregnancy prior to the manifestation of the pathology is crucial to enable timely action and improve maternal and fetal health outcomes.

Pregnancy complications such as preterm birth and early onset pre-eclampsia increase the risk in subsequent pregnancies. Conversely, a prior uncomplicated pregnancy decreases risk of complications in future pregnancies, possibly due to enduring immune alterations favoring fetal tolerance [10]. An important aspect of the immune alteration in pregnancy involves the balance of pro- and anti-inflammatory responses; typically, the immune system maintains a balance between T helper-1 (Th-1) cells, associated with cell-mediated immunity and inflammation, and T helper-2 (Th-2) cells, supporting humoral immunity and linked with anti-inflammatory responses [11, 12]. During pregnancy, the systemic maternal immune response shifts towards a more anti-inflammatory state which promotes fetal tolerance. Disruptions in this balance, particularly a switch towards a pro-inflammatory state, can lead to pregnancy complications such as pregnancy loss, pre-eclampsia, and preterm birth [11]. Even though it is important to understand these risks, we do not know much about how previous pregnancies affect certain biomarker levels related to immune regulation in the current pregnancy. However, promising research highlights the potential for early detection of preterm birth using cell-free RNA or a protein panel, as well as pre-eclampsia through a combination of sFlt-1/PlGF and ultrasound, among other methods [13–15]. A deeper understanding of the impact of previous pregnancies and the biological changes that occur before labor starts is crucial. It serves as the foundation for the development of diagnostic tests and tools aimed at improving maternal and fetal health outcomes.

Overall, the link between immune adaptation during pregnancy and obstetric complications is complex and multifaceted. While immune changes are necessary for a successful pregnancy and fetal development, they can also increase the risk of obstetric complications. Prophylactic treatment is an option for many obstetric complications, such as low-dose aspirin or progesterone. However, this requires precise risk stratification of pregnant women. Early biomarkers of later complications facilitate early interventions that can improve fetal and maternal health.

Here, we present results that build towards a deeper understanding of the immune system, the interplay between diseases and obstetric history and the immune system and its association with obstetric complications in 1049 pregnant women.

## Methods

### Study design and participants

PREGCO is a prospective cohort of pregnant women from Copenhagen University Hospital, Hvidovre, Denmark, that took place during the first coronavirus disease 2019 (COVID-19) pandemic wave in Denmark, between 4 April 2020 and 3 July 2020 [16, 17]. Participants were invited to participate at the second trimester malformation scan (gestational weeks 18–22). All pregnant women in Denmark are offered this scan and more than 90% accept. Copenhagen University Hospital Hvidovre serves approximately 12% of pregnant women in Denmark (~ 7200 births/year). Participants filled out a questionnaire including, but not limited to, pre-pregnancy body mass index (BMI), smoking, prior pregnancy outcomes and complications, and pre-existing chronic conditions. Baseline characteristics and follow-up information were obtained from the electronic health record, available throughout the whole study period. This included maternal age, gestational age at birth, sex of the child, multiple pregnancy, birth weight, results from the combined first trimester screening examination (e.g., nuchal fold thickness, pregnancy-associated plasma protein A (PAPP-A), $\beta$-human choriogonadotropin (βhCG), and crown-rump length), second trimester malformation scan (e.g., head circumference and femur length), obstetric complications (e.g., GDM, pre-eclampsia, acute cesarean section), and mode of delivery (spontaneous delivery, induction of labor, or cesarean section). See Additional file 1: Table S1 for a complete list. Furthermore, serum was collected at the 12th and 20th gestational week scans to screen for the presence of SARS-CoV-2 antibodies (IgG and IgM) using the iFlash 1800 assay [16, 17]. Serum samples from the second trimester malformation scan were also used to measure a panel of 47 inflammatory markers (described below). The study was completed and all pregnancies ended before the approval and introduction of SARS-CoV-2 vaccines in Denmark (27 December 2020).

### Comparison with the general population

The Danish Medical Birth Register (DMBR) [18] was used to compare the PREGCO cohort with all births in Denmark during the same period. DMBR, established in 1973, includes detailed data on all births in Denmark and primarily comprises data from the Danish National Patient Registry supplemented with information on pre-pregnancy BMI and smoking in the first trimester collected at the combined first trimester screening examination. We compared maternal age, pre-pregnancy BMI, smoking, parity, number of pregnancy losses, and sex of the child.

### Meso Scale Diagnostics inflammatory markers

The setup for measuring the inflammatory markers has been described by Kjerulff et al. [19]. Inflammatory markers from the second trimester malformation scan serum sample were measured using the Meso Scale Diagnostics (MSD) V-PLEX Human Biomarker 54-Plex kit, measuring 47 biomarkers involved in inflammation, chemotaxis, angiogenesis, and immune system regulation (due to poor quality observed for the TH-17 panel [19], the panel was excluded). The samples were spread across sixteen 96-well plates. We performed preprocessing and median normalization of measured intensities to minimize batch variation, as described in detail in the Additional file 1: Methods S1.

### Exposures and outcomes

We investigated the influence of pre-existing conditions (diseases and obstetric history) and diseases on biomarker levels at the second trimester malformation scan. This included smoking during pregnancy, number of prior live births, number of prior pregnancy losses, polycystic ovary syndrome (PCOS), endometriosis, inflammatory bowel disease (IBD), GDM in the current or previous pregnancy, pre-eclampsia in a prior pregnancy, vaginal bleeding in early pregnancy, use of assisted reproductive technology (ART, divided into intrauterine insemination (IUI) and in-vitro fertilization (IVF)), and COVID-19 infection in the current pregnancy. The COVID-19 assay and cut-offs have been previously described by Freiesleben et al. and Egerup et al. [16, 17]. Second, we also investigated the association between inflammatory markers and common obstetric complications, namely GDM, pre-eclampsia, gestational duration, acute cesarean section, and the ratio of birth weight to gestational duration (Additional file 1: Table S1). To reduce redundancy in the statistical analysis, we identified highly correlated markers using the Hobohm II algorithm [20]. We tested multiple thresholds and found that a Spearman correlation cut-off of 0.5 sufficiently removed redundant markers (Additional file 1: Fig. S1). For further details, see Additional file 1: Methods S1.

### Machine learning models to predict obstetrical complications

We evaluated the predictive value of the clinical measures and the MSD biomarkers using two machine learning models, namely a logistic regression model with an L1 penalty and LightGBM [21]. Missing values were imputed using the mean and mode for continuous and categorical variables, respectively, and scaled for the logistic regression model. Imputation and scaling were done strictly on training data. LightGBM is a gradient-boosted model that natively handles missing values and categorical data, and scaling is not needed. The logistic regression model with an L1 penalty provides a sparse set of features, enabling interpretability, but cannot detect non-linear patterns and interactions unless explicitly defined. Nor does the logistic regression handle missing values. In contrast, LightGBM, a fast implementation of a gradient-boosted model, automatically identifies non-linear patterns and interactions and can handle missing data, albeit at the cost of interpretability. Other methods, such as neural networks, typically underperform tabular data of limited size, and kernel methods do not scale well.

As features, we used the 47 MSD inflammatory markers, age, pre-pregnancy BMI, previous live births, fetal abdominal circumference, fetal abdominal diameter, fetal head circumference, fetal femur length, PAPP-A multiples of medians (MoM), βhCG MoM, and the difference between gestational age measured from crown-rump length and last menstruation at the combined first trimester screening examination.

The models’ generalizability was evaluated using a nested cross-validation (CV) procedure. Here, the nested CV involved an outer loop for model evaluation (interval validation) and an inner loop for hyperparameter optimization (development data). The outer loop was a five-fold stratified CV, and the inner loop was a fivefold stratified CV repeated five times. Hyperparameters were optimized using the Optuna [22] framework with the Tree-structured Parzen Estimator algorithm. Additional file 1: Table S2 provides the ranges of hyperparameters that were explored. A total of 500 hyperparameter combinations were evaluated across the inner CV loops to identify the most effective configuration for the model. The models were optimized to minimize the binary cross entropy (BCE). We selected the model with the lowest average inner CV BCE. We evaluated the area under the receiver operating characteristic curve (ROC-AUC) and the area under the precision-recall curve (AUPRC) by averaging the scores from the outer CV. Feature importance was evaluated using Shapley additive explanations (SHAP) on the hold-out outer fold. The ROC-AUC ranges from 0.5 (random) to 1.0 (perfect). However, in cases of severe class imbalance, the ROC-AUC may be biased. Therefore, we also evaluated the AUPRC. The AUPRC ranges from 0 to 1. However, the baseline value (corresponding to a random classifier) is equivalent to the prevalence. 95% Confidence intervals for the ROC-AUC and AUPRC were calculated using a bootstrap approach, with 1000 repetitions. We used the linear (for logistic regression) or tree (for LightGBM) explainer algorithm. Results were visualized as the mean absolute SHAP values. Machine learning pipelines were implemented as a snakemake workflow (v 7.19.0), using elements from Optuna (v 3.2.0), scikit-learn (v 1.2.1), and SHAP (v 0.42.0) [22–24].

### Statistical analysis

Baseline characteristics were summarized as mean (standard deviation, SD) or median (interquartile range, IQR), where appropriate. Baseline characteristics were compared to data from the DMBR for all births in 2020 using a *Z*-test or ${\chi }^{2}$ test. Heatmaps were created by calculating pairwise Spearman’s correlation coefficients. Clustering was done using hierarchical complete clustering. Only MSD biomarkers were used for clustering.

The association between prior existing conditions and inflammatory marker levels and birth weight-gestational age ratio was estimated using a Bayesian robust linear regression model to accommodate outliers [25]. Effects sizes are reported as deviations from the mean.

The association between inflammatory markers and GDM, pre-eclampsia, gestational duration, acute cesarean section, severe postpartum hemorrhage (PPH), and any complication was estimated using a cause-specific Bayesian time-to-event model, in which the baseline hazard was modeled using an M-spline [26]. Effect sizes are reported as the log hazard rate. Women lost to follow-up were censored at their last contact with the hospital. All analyses were adjusted for age, pre-pregnancy BMI, and gestational age at enrollment. The association between prior conditions and inflammatory markers was further adjusted for the ultrasound estimated gestational age at the first trimester risk assessment scan. Obstetric outcomes were adjusted for outcome-specific variables that were identified based on expert and literature review. Missing values were imputed using multiple imputation as implemented in MICE [27], with predictive mean matching. Chains were run for 100 iterations and five imputation data sets were created. Each imputed data set was analyzed separately in the Bayesian models, and the posteriors were then combined. Conservative prior distributions were used for the Bayesian models, providing a regularizing effect.

Estimates are reported as the median and the 95% credible interval (bCI) unless otherwise stated. See Additional file 1: Methods S1 for a more detailed description of the methods and models. All analysis was performed in R (v 4.0.0). All models were fitted using rstanarm (v 2.21.3) [26] or brms (v 2.18.0) [25], and results were plotted using ggplot (v 3.4.2) and associated packages available through the comprehensive R archive network (CRAN, https://cran.r-project.org/).

## Results

### Cohort characteristics

**Fig. 1:**
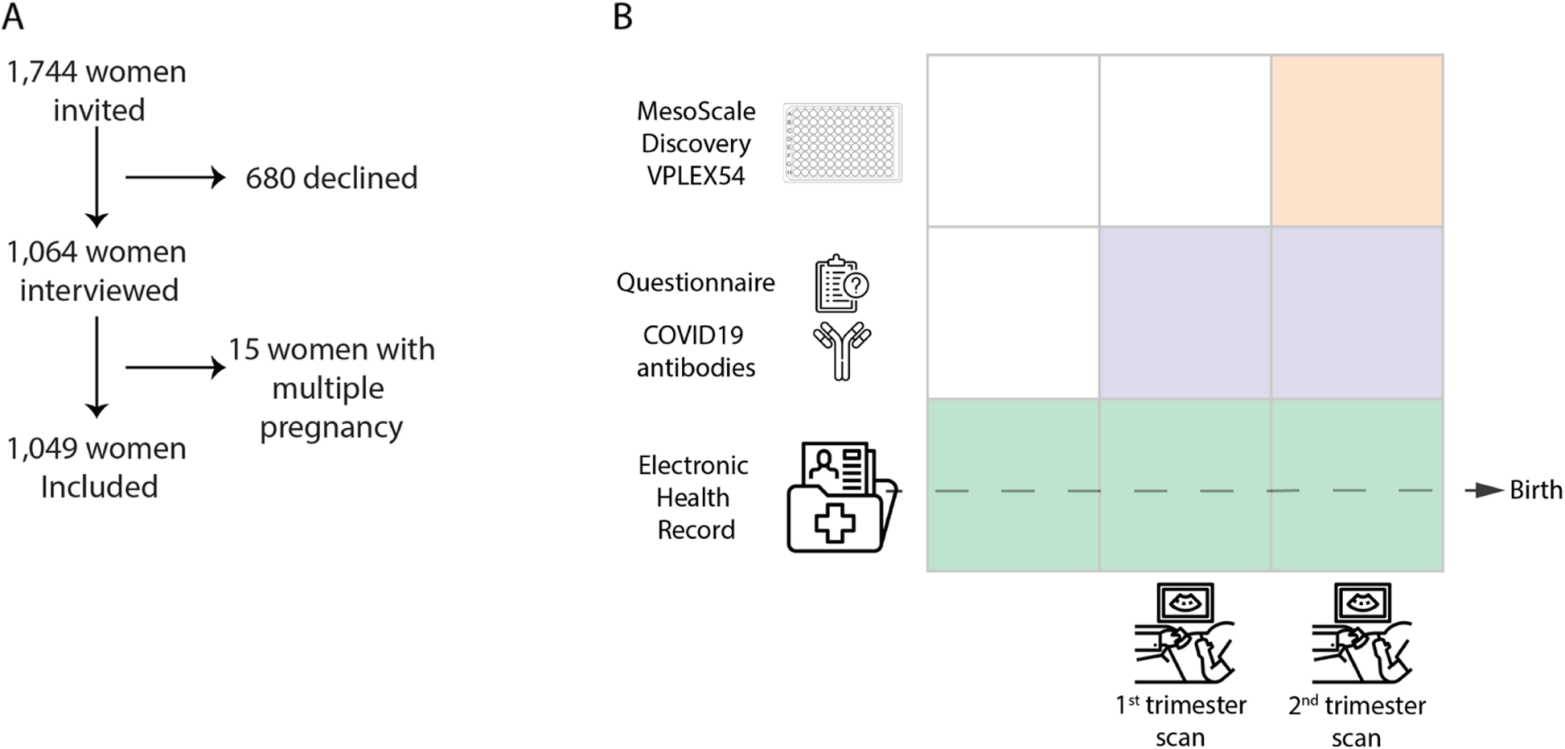
**a** Flowchart for inclusion. **b** Timepoints for data collection. Data from the electronic health record was obtained throughout the pregnancy and postpartum

A total of 1064 women were enrolled in this study during the second trimester malformation scan at 20 weeks. The cohort constitutes 75% of all pregnant women asked to participate (flowchart shown in Fig. 1A). Of these, 15 were not included due to multiple pregnancy, and 18 were lost to follow-up due to a change of hospital not using the EPIC electronic platform for medical records or home delivery. The baseline characteristics for participants are shown in Table 1. We identified 26 women who received prophylactic acetylsalicylic acid (ASA) treatment prior to inclusion. Compared to all births in Denmark in 2020, maternal age, pre-pregnancy BMI, and the number of previous live births are within the expected range, albeit they are on average 1 year older, 0.7 BMI units lower, and have a higher frequency of primipara and prior pregnancy losses (Additional file 1: Table S3). Thirty-three women (3.1%) had elevated SARS-CoV-2 IgG antibody levels indicating COVID-19 infection prior to the second trimester malformation scan. During the study, 369 (34.7%) women experienced at least one of the complications severe PPH (12.2%), preterm birth (11%), GDM (8.2%), pre-eclampsia (4.3%), or acute cesarean Sect. (11.8%) after enrollment (Table 2).

**Table 1:** Baseline characteristics for the 1049 women included in the PREGCO cohort

**Variable**		**PREGCO (*n* = 1049)**
Maternal age, years*		31.7 (4.5)
Gestational age at inclusion, days**		141 (140–143)
Pre-pregnancy BMI, kg/m^2^*		24.1 (4.8) (na = 9)
Smoking in pregnancy		40 (3.8%)
Acetylsalicylic acid treatment, prior to inclusion		26 (2.5%)
Parity	0	576 (54.9%)
	1	375 (35.7%)
	2	81 (7.7%)
	3 +	17 (1.6%)
Number of pregnancy losses	0	810 (77.2%)
	1	194 (18.5%)
	2	29 (2.8%)
	3	16 (1.5%)
Sex of child, male		521 (50.5%) (na = 18)
$\beta$ HCG***		1.2 (0.8) (na = 35)
PAPP-A***		1.3 (0.8) (na = 35)
COVID-19 antibodies		34 (3.2%)
Conception method	Spontaneous	944 (88.7%)
	Assisted reproductive technology (IVF or IUI)	120 (11.3%)
Prior existing chronic conditions	PCOS	23 (2.2%)
	Diabetes mellitus (type 1 or type 2)	2 (0.2%)
	Endometriosis	9 (0.8%)
Complications in previous pregnancy	Preterm birth	19 (1.8%)
	Pre-eclampsia	24 (2.3%)
	PPH (> 500 mL blood loss)	20 (1.9%)
	GDM	12 (1.1%)

Symbols: * mean (sd); ** median (IQR); *** multiple of medians (MoM). Missing values denoted in na. If no mention of missing values, the value is complete

*Abbreviations*: *BMI*, body mass index, *GDM*, gestational diabetes; *IUI*, intrauterine insemination; *IVF*, in vitro fertilization; *PCOS*, polycystic ovary syndrome; *PPH*, postpartum hemorrhage

**Table 2:** Outcome characteristics

**Variable**		**PREGCO**
Birth weight (g)*		3499 (554)
Gestational age (days)**		280 (273; 287)
Preterm (< 37 + 0)		116 (11%)
Birth weight/gestational age**		12.6 (1.8)
Severe postpartum hemorrhage (PPH)		130 (12.2%)
Pre-eclampsia		46 (4.3%)
Gestational diabetes mellitus (GDM)		87 (8.2%)
Mode of delivery	Spontaneous vaginal birth	634 (60.7%)
	Acute cesarean section	126 (11.8%)
	Elective cesarean section	95 (8.9%)
	Induced birth	235 (22.1%)
Any complication	(Severe PPH, preterm birth, GDM, pre-eclampsia, acute cesarean section)	369 (34.7%)

Symbols: * mean (sd); ** median (IQR)

*Abbreviations*: *GDM*, gestational diabetes mellitus; *PPH*, postpartum hemorrhage

### Biomarker profile clustering

**Fig. 2:**
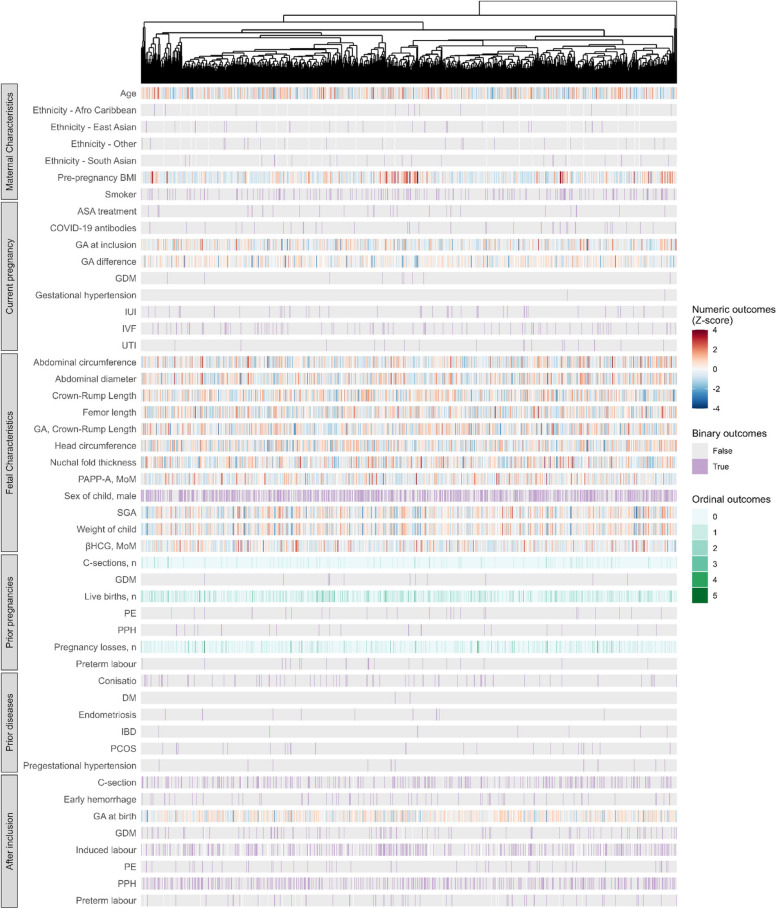
Heatmap of phenotypes across participants using hierarchical clustering based on MSD biomarkers. Phenotypes are divided into maternal characteristics, pregnancy-related outcomes for the current pregnancy (observed before or at inclusion), fetal characteristics, pregnancy-related outcomes for prior pregnancies, prior diseases to the current pregnancy, and phenotypes observed after inclusion. ASA, acetylsalicylic acid; BMI, body mass index; C-section, cesarean section; DM, diabetes mellitus; GA, gestational age; GDM, gestational diabetes mellitus; IBD, inflammatory bowel diseases; IUI, intrauterine insemination; IVF, in vitro fertilization; MoM, multiples of medians; PCOS, polycystic ovary syndrome; PE, pre-eclampsia; PPH, postpartum hemorrhage; SGA, small for gestational age; UTI, urinary tract infection

Clustering analysis did not reveal any strong relationships between the women’s inflammatory profiles and their prior conditions, diseases, or later complications (Fig. 2, Additional file 1: Fig. S2A). When we examined the pairwise correlations between markers, we found that they did not necessarily cluster by panel or marker group (Additional file 1: Fig. S2B). Instead, we observed one large cluster and some minor clusters. The largest cluster, with the strongest correlations, consisted of MCP-4, TARC, IL-8, VEGF-C, and IL-7. MCP-4, TARC, IL-8, VEGF-C, and IL-7 are all cytokines linked to various inflammatory diseases.

The strongest anti-correlation observed was between Flt-1 and VEGF (− 0.58, 95% CI − 0.62; -0.53, Pearson correlation). By binding VEGF and blocking the membrane-bound receptors, soluble Flt-1 functions as a naturally occurring antagonist of VEGF. Soluble Flt-1 also binds placental growth factor (PlGF) which plays a crucial role in the growth and development of blood vessels, particularly during pregnancy and fetal development [28]. Dysregulation of the interaction between Flt-1 and VEGF has been linked to various diseases and disorders, including cancer and cardiovascular disease.

### Pre-existing conditions, obstetrical history, and maternal characteristics

**Fig. 3:**
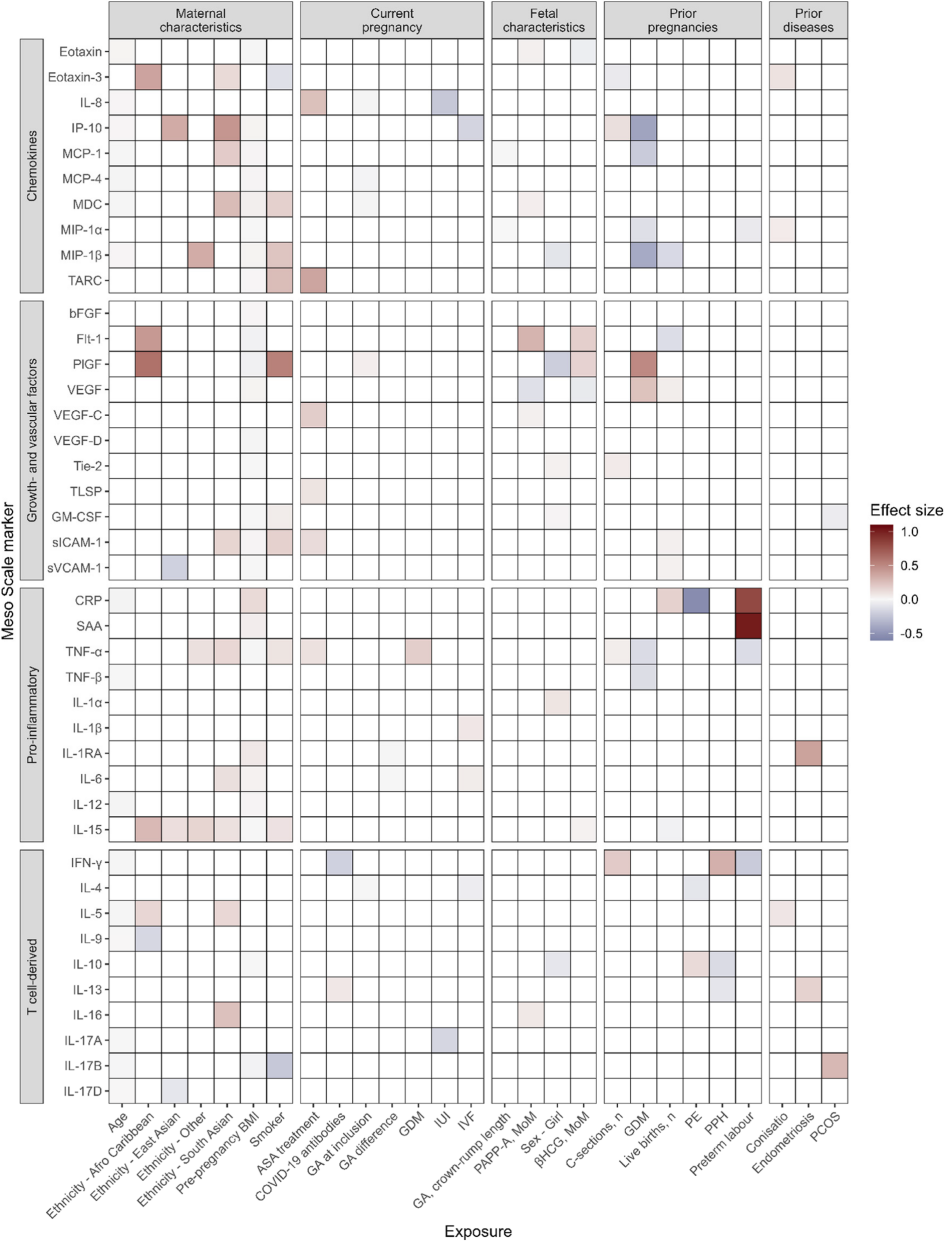
Previous pregnancies and pre-existing conditions effects on inflammatory markers. Effect size is increase or decrease in standard deviations. Only associations where the 95% Bayesian credible interval does not include zero are shown. ASA, acetylsalicylic acid; BMI, body mass index; GA, gestational age; GDM, gestational diabetes mellitus; IUI, intrauterine insemination; IVF, in vitro fertilization; MOM, multiples of medians; PCOS, polycystic ovary syndrome; PE, pre-eclampsia; PPH, postpartum hemorrhage

A number of previously existing conditions correlated to the measured inflammatory markers. However, there was no widespread pleiotropy, i.e., each exposure had its own biomarker signature (Fig. 3). A majority of biomarkers were affected by maternal age (17/47) and pre-pregnancy BMI (27/47). Conditions altering expression levels included chronic diseases (endometriosis, PCOS), complications in prior pregnancies (pre-eclampsia, GDM, preterm birth), and COVID-19. COVID-19 was associated with a change in expression of two inflammatory markers, IFN-γ and IL-13. Smoking in the current pregnancy had a profound effect on ten biomarkers, including GM-CSF ($\beta$=0.05, 95% bCI 0.01; 0.09). GM-CSF is a known regulator of fetal growth and the association between smoking and increased GM-CSF levels is hypothesized to be through an activation of the epidermal growth factor receptor (EGFR) signaling pathway [29]. GM-CSF was also upregulated in pregnancies with a female child, albeit to a lower degree ($\beta$ = 0.02, 95% bCI 0.00; 0.03). A female child was also associated with decreased levels of PlGF, MIP-1β, and IL-10 and increased levels of IL-1ɑ and Tie-2. Altered CRP levels were associated with complications occurring in prior pregnancies, e.g., pre-eclampsia ($\beta$ = − 0.53, 95% bCI − 0.98; − 0.09) and preterm birth ($\beta$ = 0.82, 95% bCI 0.33; 1.3), even when accounting for prophylactic acetylsalicylic acid treatment. Prophylactic acetylsalicylic acid treatment increased the relative level of TARC, IL-8, VEGF-C, siCAM1, TNF-α, and TLSP. The number of previous live births was also associated with lower CRP levels, which could explain part of the mechanism behind the reduced risk of pre-eclampsia ($\beta$ = 0.17, 95% bCI 0.07; 0.28).

TNF-α levels were increased in women with GDM prior to the second trimester malformation scan ($\beta$ = 0.18, 95% bCI 0.01; 0.36) and decreased in women with GDM in prior pregnancies ($\beta$ = − 0.14, 95% bCI − 0.23; − 0.04). TNF-α is a marker of insulin resistance in pregnancy [30], and our findings indicate that insulin resistance is not affected in the longer term in GDM pregnancies. This suggests that increased TNF-α levels can be attributed to the acute pathogenic process.

Ethnicity had a major influence on the variation of inflammatory marker levels, such as eotaxin-3, IL-15, IL-16, and IP-10, among others (Fig. 3). These findings highlight the need for more research on the role of ethnicity in pregnancy complications and the underlying mechanisms. This research could inform the development of personalized interventions to reduce the risk of adverse pregnancy outcomes by taking into account reference ranges that may be specific to ethnic groups.

### Later obstetric complications

**Fig. 4:**
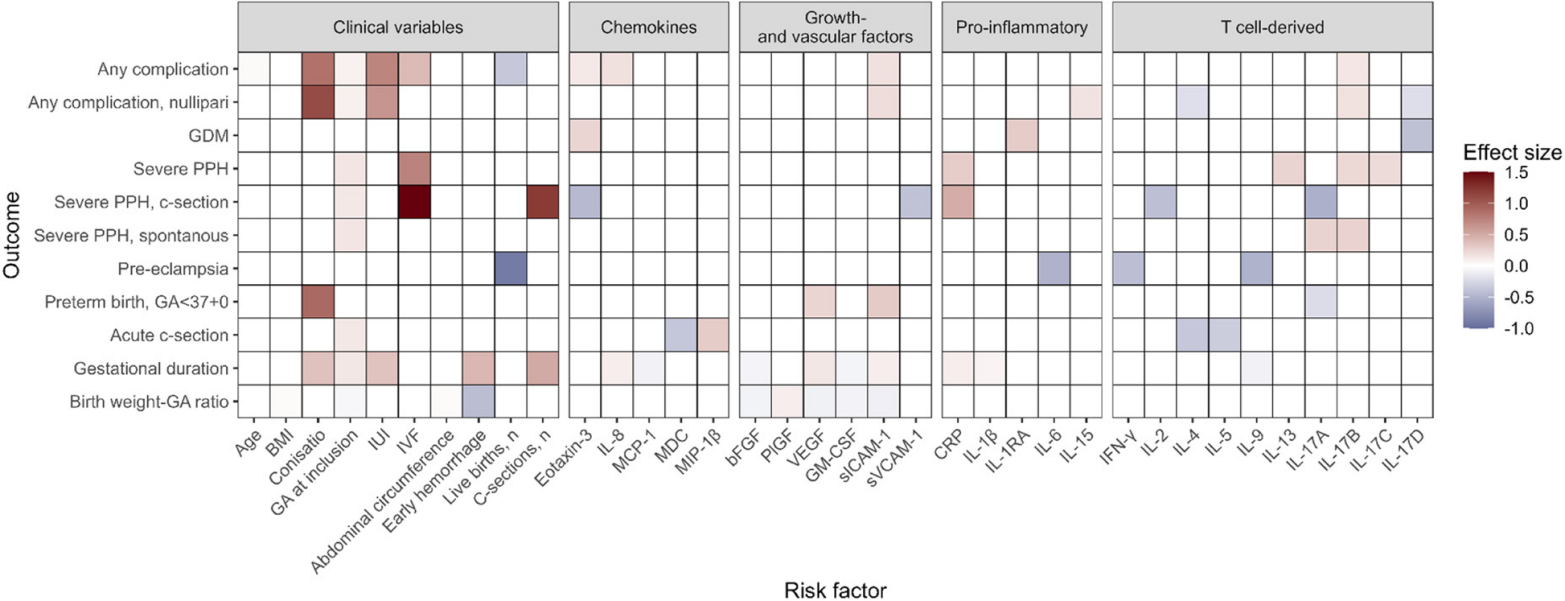
Associations between markers and later obstetrical outcomes. The effect size is the log-hazard rate for all outcomes, except birth weight-gestational age ratio (BWGA). The effect of biomarkers on BWGA is a change in percentile from the 50th percentile. Only associations where the 95% Bayesian credible interval does not include zero are shown. BMI, body mass index; GA, gestational age; IUI, intrauterine insemination; IVF, in vitro fertilization

Following similarity reduction, we estimated the association between 41 markers and 11 different outcomes (Fig. 4). We found a number of unique markers that were associated with each condition. Increasing levels of IL-6 was associated with decreasing risk of pre-eclampsia (hazard rate (HR) = 0.59, 95% bCI 0.31; 0.97). Prior evidence clearly indicates an association, but the direction of effect is less clear [31, 32]. Acute cesarean section had four associated markers (IL-4, IL-5, MDC, MIP-1β) none of which were associated with any of the other adverse outcomes. For the birth weight to gestational age ratio, we found five associated markers: bFGF, GM-CSF, PlGF, sICAM-1, and VEGF. The most profound effect on birth weight to gestational age ratio was seen for vaginal bleeding in early pregnancy, which led to an estimated decrease in percentile of − 0.16 (95% bCI − 0.06; − 0.25). This means that a woman experiencing early hemorrhage would be expected to be in the 34th percentile (95% bCI 25; 44). In comparison, PlGF, the inflammatory marker with the largest effect, was associated with an increased percentile of 0.04 (95% bCI 0.01; 0.07) per standard deviation.

GDM was associated with IL-1RA (HR = 1.35, 95% bCI 1.04; 1.8), IL-17D (HR = 0.66, 95% bCI 0.49; 0.89), and eotaxin-3 (HR = 1.27, 95% bCI 1.11; 1.43). IL-1RA has previously been shown to associate with GDM and has been suggested as a biomarker useful for diagnosing GDM as a complement to blood glucose measurements as well as to identify GDM patients who are at risk of developing postpartum diabetes [33]. IL-17D is associated with incident type 2 diabetes and progression from normoglycemia to type 2 diabetes [34]. GDM is associated with several chemokines in the protein family where eotaxin-3/CCL26 belongs [35, 36].

CRP (HR = 1.34, 95% bCI 1.04; 1.74), IL-13 (HR = 1.29, 95% bCI 1.06; 1.54), IL-17B (HR = 1.25, 95% bCI 1.02; 1.53), and IL-17C (HR = 1.23, 95% bCI 1.01, 1.49) were all associated with severe PPH. However, none of the effects had a magnitude similar to IVF (HR = 2.10, 95% bCI 1.18; 3.66).

### Prediction of obstetric complications

We evaluated the prognostic potential of clinical characteristics combined with MSD biomarkers in predicting five pregnancy-related conditions. The performance of our model was assessed using ROC-AUC values on the development data (inner CV) ranging from 0.610 to 0.697, and similar values were obtained in the interval validation (outer CV), ranging from 0.584 to 0.715. These results indicate that our model generalizes well to new data. The AUPRC values were consistently better than random guessing, as shown in Additional file 1: Table S5.

**Fig. 5:**
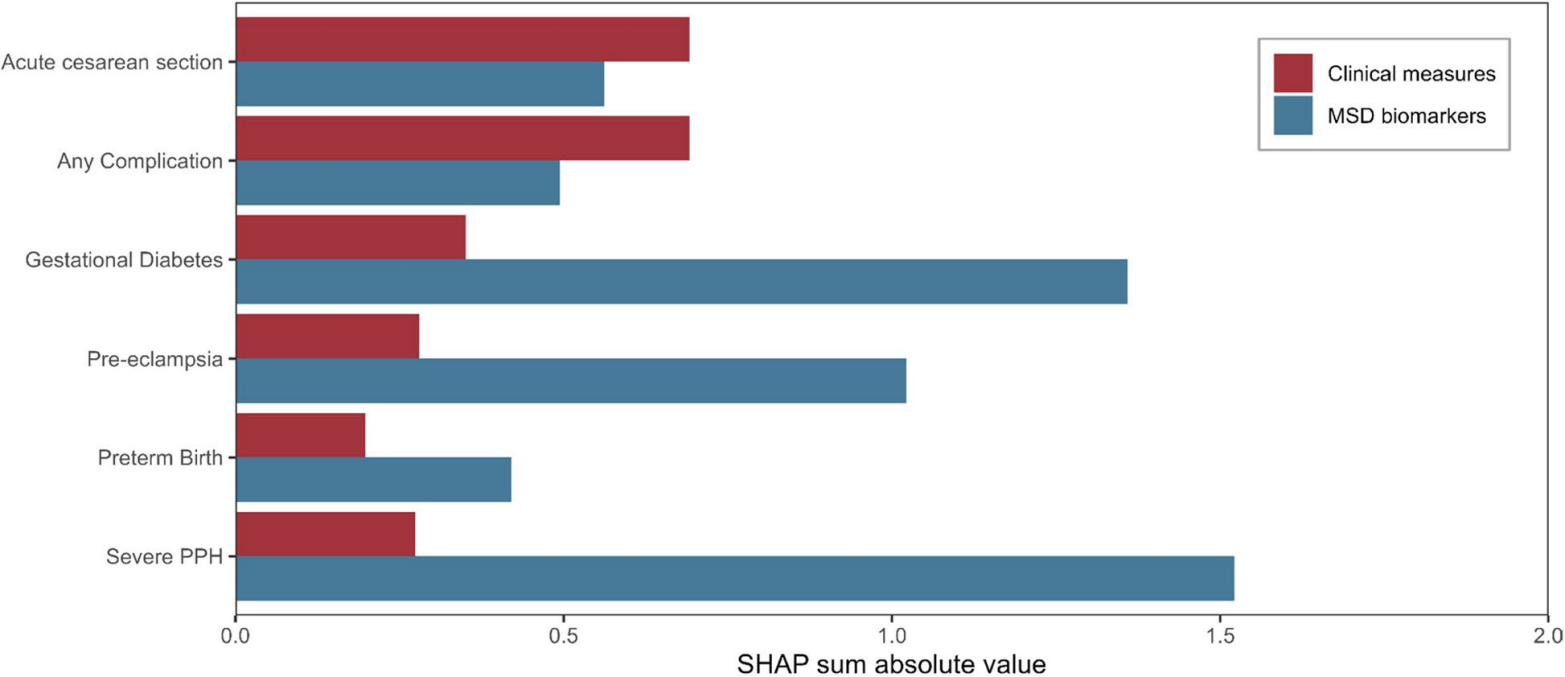
Summarized SHAP values for each feature category (red = clinical measure, blue = MSD biomarker) across all outcomes investigated in the prognostic model. PPH, postpartum hemorrhage

Among the pregnancy-related conditions analyzed, pre-eclampsia, GDM, and PPH were predicted most accurately using the MSD biomarkers (Fig. 5). Specifically, GDM and pre-eclampsia displayed ROC-AUC values of 0.708 (95% CI: 0.644–0.766) and 0.672 (95% CI: 0.580–0.758), respectively, with corresponding AUPRC values of 0.176 (95% CI: 0.126–0.238) and 0.073 (95% CI: 0.042–0.115).

**Fig. 6:**
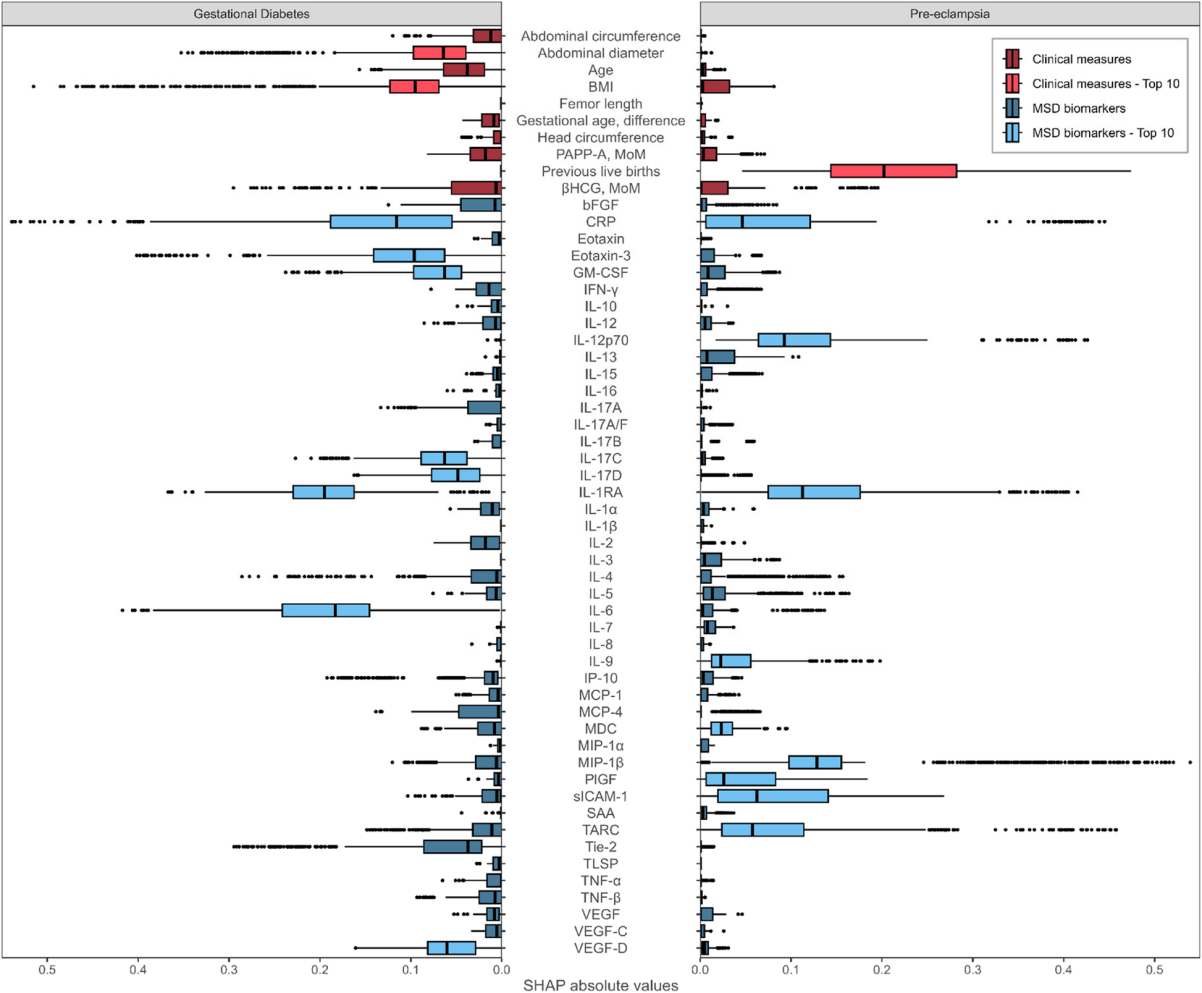
SHAP values for all prognostic features for gestational diabetes mellitus (GDM) and pre-eclampsia, respectively. The top ten features with the highest median value for each outcome are indicated with light colors. Only IL-1RA and CRP were part of both top ten lists. Features are colored based on the feature category as clinical measure (red) or MSD biomarker (blue). BMI, pre-pregnancy body mass index; MoM, multiple of medians; SHAP, Shapley additive explanation

The SHAP analysis further confirmed the classification of IL-1RA and eotaxin-3 as potential predictive biomarkers for GDM (Fig. 6). Additionally, the analysis highlighted the significance of IL-6 and CRP, two pro-inflammatory markers that were initially overlooked in the preliminary association analysis but have been previously suggested as predictive for GDM [37–39].

Although the exploratory analysis yielded only a single probable biomarker for pre-eclampsia, our predictive modeling emphasized the combined significance of MIP-1β, IL-1RA, and IL-12p70. These biomarkers collectively had a greater impact than conventional risk factors such as age and BMI. Notably, the number of prior live births remained the most influential predictor (Fig. 6).

The remaining models had a low ability to discriminate and were not investigated further.

## Discussion

Here, we present the biomarker profile of a large population-representative cohort of pregnant women around the 20th gestational week. We show that pregnancy has long-lasting effects and that the molecular level rewiring takes place at very early stages in the pregnancy, prior to the manifestation of complications at a clinically detectable level. Early changes in biomarkers were associated with the development of obstetric outcomes up to 22 weeks later. Moreover, prior live births, complications from prior births, and smoking affect circulating biomarker levels, which may have a direct effect on complications and fetal growth. Machine learning models highlighted the joint significance of inflammatory markers, albeit the performance is not yet adequate for clinical deployment.

PREGCO is a prospective cohort recruited at a single hospital. Enrollment was high, above 61%, and the prospective nature of the design limits bias, as seen in case–control studies. The cohort was generally comparable to other pregnancies in Denmark in the same time period. The difference in the frequency of pregnancy loss can most likely be attributed to an underreporting in the Danish National Patient Registry. The cohort was enrolled during a time of lowered activity in Denmark due to restrictions on both professional and private social interactions, hence the COVID-19 prevalence was low at this time. Furthermore, enrollment took place when only the alpha COVID-19 variant was present, and later variants may have different effects on the immune system [40, 41]. Furthermore, the testing regime and willingness to seek medical attention could have been different due to the COVID-19 pandemic. Relative to the sample size, the number of biomarkers investigated was large and some were highly correlated. We pruned highly correlated markers and utilized Bayesian models with conservative priors to provide regularization to the estimates to mitigate this and investigate issues of collinearity. We evaluated the generalization error of the machine learning models using a nested cross-validation approach, which yielded conservative estimates. However, we cannot rule out that some effects could not be detected due to the sample size, the large number of variables, and conservative Bayesian priors. Lastly, while this study offers valuable insights, it is essential to acknowledge that the correlation between laboratory data and clinical outcomes, while suggestive, can be complex and influenced by numerous factors not fully explored here.

Pregnancy is a complex process that involves many changes in the body, including challenges to the immune system. These changes are necessary to allow the developing fetus to grow and thrive, but they can also increase the risk of obstetric complications. For instance, GM-CSF, a known regulator of fetal growth, was upregulated in pregnancies with a female child or smoking. The association between smoking and increased GM-CSF levels is hypothesized to be through an activation of the EGFR signaling pathway [29]. Furthermore, preterm birth or pre-eclampsia in a previous pregnancy was associated with lower levels of CRP. This is most likely medically induced due to preventive treatment with aspirin and progesterone, respectively.

Supporting previous literature, our study identified associations between VEGF and PlGF with the birth weight/gestational age ratio. VEGF and PlGF are key regulators of angiogenesis and placental development. Disruptions in their levels can impair placental function and fetal growth, leading to a lower birth weight/gestational age ratio. This may result from insufficient placental blood flow due to altered VEGF and PlGF signaling [42, 43].

The balance between pro- and anti-inflammatory cytokines is crucial for successful placentation. Pro-inflammatory cytokines, such as TNF-$\alpha$, IL-1, and IL-6, play a role in the recruitment and activation of immune cells at the implantation site, which is essential for the establishment of a functional placenta [44]. IL-6 is involved in the regulation of angiogenesis, decidualization, and immune cell migration during early pregnancy, suggesting a crucial role for IL-6 in placentation [45]. Likewise, elevated levels of IL-17A have been associated with preterm labor [46]. One proposed mechanism for this is IL-17A’s ability to promote pro-inflammatory cytokines and chemokines that can affect the development of the fetal-placental interface, especially together with TNF-$\alpha$ [46]. In our study, the pro-inflammatory cytokines of the IL-17-family were associated with disorders that have been linked with a negatively altered placentation, such as postpartum hemorrhage or preterm birth. In our study, we observed associations between CRP, IL-13, IL-17B, and IL-17C, with severe PPH. It is important to note, however, that markers like CRP and IL-6 are nonspecific indicators of inflammation and their elevation can be triggered by a variety of factors. CRP is a non-specific acute-phase reactant produced by the liver in response to inflammation, infection, and tissue damage. Similarly, IL-6, a cytokine involved in the regulation of immune responses, can be elevated due to various conditions. While these biomarkers can signal disturbances in the maternal body, their direct association with specific pregnancy complications requires cautious interpretation. IL-13 is a Th-2 cell cytokine involved in the regulation of immune response and tissue repair, whereas IL-17B and IL-17C are members of the IL-17 family of cytokines that are involved in the regulation of inflammation and immunity. Alterations in these biomarkers may affect the normal balance between pro- and anti-inflammatory factors and disrupt the physiological processes during and after delivery, leading for example to an increased risk of PPH. In the third trimester, it has previously been shown that higher concentrations of maternal plasma cytokines, including IL-17, were associated with an increased risk of atonic PPH [47, 48]. This suggests that elevated levels of these inflammatory cytokines may impair uterine contractility and hemostatic balance, exacerbating bleeding risk. In our study, the biomarkers showed these associations to the outcome around gestational week 20, making them potential candidates for clinical use in early detection and prevention.

GDM is associated with an increased risk of maternal and fetal morbidity and mortality as well as an increased risk of developing type 2 diabetes later in life. The precise mechanisms underlying the development of GDM are not yet fully understood, but evidence suggests that a complex interplay of genetic and environmental factors is involved. In our study, eotaxin-3, IL-1RA, and IL-17D were associated with GDM. All three cytokines have been implicated in the regulation of immune responses, inflammation, and metabolic regulation. For example, elevated levels of eotaxin-3 have been observed in pregnant women with pre-existing diabetes [49]. Antagonizing the pro-inflammatory cytokine IL-1, IL-1RA is an anti-inflammatory cytokine [50]. The interplay of IL-1 and IL-1RA and their dysregulation appear to be related to type 2 diabetes and GDM [33, 51]. However, study results are diverging, as for example one study has shown that the IL-1RA levels in GDM patients are considerably lower than those of controls [33], whereas another biomarker study showed inconsistence with our results, that elevated IL-1RA are associated with GDM [36]. In summary, our data show that the levels of these cytokines are altered in women developing GDM later in pregnancy and that the balance of pro- and anti-inflammatory cytokines is necessary to maintain a normal glucose metabolism throughout pregnancy.

We identified MIP-1β, IL-1RA, and IL-12p70 to have a large predictive value for pre-eclampsia. MIP-1β is an important chemokine in early placentation promoting trophoblast migration [48], and higher expression or concentration of MIP-1β has been found under pre-eclampsia in the uterine decidua [52], the placenta [53], and third trimester serum [54]. Moreover, placental microvesicles from pre-eclamptic placentas have been shown to have a greater potential to induce MIP-1β expression in peripheral blood mononuclear cells as compared to microvesicles from normal pregnancy placentas [55]. IL-12p70 is a pro-inflammatory interleukin and has been shown to Th1 differentiation and IFN-γ production, contributing to a pro-inflammatory shift and impaired placentation [56, 57]. However, previous studies of low sample sizes showed mixed results for the association between IL-12p70 and pre-eclampsia [54, 58–60]. The majority of the pre-eclampsia cases were late-onset, and thus the findings may not generalize to cases of early onset pre-eclampsia.

Additionally, the study highlights the importance of considering multiple biomarkers in assessing risk of obstetric complications, as each biomarker was associated with only one or a few of the outcomes. This suggests that a combination of biomarkers may be necessary to accurately identify women who are at an increased risk of developing complications. Our study also supports the notion that one blood sample, at the second trimester, in which a panel of biomarkers are measured, can be used as input for multiple machine learning models that each provide a risk assessment for very heterogeneous conditions in pregnancy.

Once they arise, many obstetric complications cannot be reversed or treated. Perinatal medicine therefore aims to identify high-risk populations early on, to ideally use therapies to reduce unfavorable maternal and fetal outcomes [61]. Dietary adaptations and insulin for GDM, antepartum fetal monitoring for stillbirth, acetylsalicylic acid for pre-eclampsia, and progesterone for preterm delivery are a few examples of such therapies that have been proposed in high-risk populations [62–67]. As such, it has been proposed that the typical care pyramid should be reversed, with the primary attention shifting to the early rather than later stages of pregnancy [61].

## Conclusions

Our study makes two novel contributions to the literature: First, we demonstrate that pregnancy complications can have molecular signatures that persist and impact subsequent pregnancies. Second, we reveal distinct molecular profiles associated with different types of pregnancy complications.

The presented biomarkers and biomarker combinations can potentially be used to further improve this pyramid of care by providing high-quality care to the patients at risk.

## Supplementary Information

Additional File 1: Methods S1, Figs. S1-S3, Tables S1-S6. Methods S1. Additional information on data preprocessing and modeling. Fig. S1. Pruning by the Hobohm II algorithm. Fig. S2. Correlation between MSD markers. Fig. S3. PCA plots for the six MSD panels. Table S1. Outcome descriptions, definitions, adjustments and exclusion criteria. Table S2. Hyperparameter values. Table S3. Comparison of PREGCO and the Danish Medical Birth Registry (DMBR). Table S4. Variables and sources. Table S5. Results from cross-validation of machine learning models. Table S6. Birth weight-gestational duration ratio parameters estimated from the Danish Medical Birth Registry (DMBR).

## Data Availability

No datasets were generated or analysed during the current study. The analyses in this study were conducted using standard open-source software and tools. The procedures for data preparation and analysis are described comprehensively in the “Methods” section to enable replication using similar standard tools. Analysis code can be provided upon reasonable request to the corresponding authors.

## Abbreviations

ART: Assisted reproductive technology
ASA: Acetylsalicylic acid
AUPRC: Area under the precision-recall curve
βhCG: $\beta$-Human choriogonadotropin
BCE: Binary cross entropy
bCI: Credible interval
BMI: Body mass index
COVID-19: Coronavirus disease 2019
CRAN: Comprehensive R archive network
CV: Cross-validation
DMBR: Danish Medical Birth Register
EGFR: Epidermal growth factor receptor
GDM: Gestational diabetes mellitus
HR: Hazard rate
IBD: Inflammatory bowel disease
IQR: Interquartile range
IUI: Intrauterine insemination
IVF: In-vitro fertilization
MERS-CoV: Middle East respiratory syndrome
MoM: Multiples of medians
MSD: Meso Scale Diagnostics
PAPP-A: Pregnancy-associated plasma protein A
PCOS: Polycystic ovary syndrome
PlGF: Placental growth factor
PPH: Postpartum hemorrhage
ROC-AUC: Area under the receiver operating characteristic curve
RSV: Respiratory syncytial virus
SARS-CoV: Severe acute respiratory syndrome
SD: Standard deviation
SHAP: Shapley additive explanations
Th-1: T helper-1 cells
Th-2: T helper-2 cells
