# Supplementary material for: Immune changes in pregnancy: associations with pre-existing conditions and obstetrical complications at the 20th gestational week—a prospective cohort study
Source: BMC Med. 2024 Dec 18;22:583. doi: 10.1186/s12916-024-03797-y (PMC11657209; doi:10.1186/s12916-024-03797-y)
Supplement: Supplementary file 1 — Additional File 1: Methods S1, Figs. S1-S3, Tables S1-S6. Methods S1. Additional information on data preprocessing and modeling. Fig. S1. Pruning by the Hobohm II algorithm. Fig. S2. Correlation between MSD markers. Fig. S3. PCA plots for the six MSD panels. Table S1. Outcome descriptions, definitions, adjustments and exclusion criteria. Table S2. Hyperparameter values. Table S3. Comparison of PREGCO and the Danish Medical Birth Registry (DMBR). Table S4. Variables and sources. Table S5. Results from cross-validation of machine learning models. Table S6. Birth weight-gestational duration ratio parameters estimated from the Danish Medical Birth Registry (DMBR). [file 12916_2024_3797_MOESM1_ESM.pdf]

Additional file 1

Title: Immune Changes in Pregnancy: Associations with Pre-existing Conditions and Obstetrical Complications at the 20th Gestational Week - A Prospective Cohort Study

David Westergaard[1,2,3]#, Agnete Troen Lundgaard[1]#, Kilian Vomstein[3], Line Fich [3], Kathrine Vauvert Römmelmayer Hviid[3], Pia Egerup[3], Ann-Marie Hellerung Christiansen[3], Josefine Reinhardt Nielsen[3], Johanna Lindman[3], Peter Christoffer Holm[1], Tanja Schlaikjær Hartwig[3], Finn Stener Jørgensen[3,6], Anne Zedeler[3], Astrid Marie Kolte[3], Henrik Westh[4,6], Henrik Løvendahl Jørgensen[5,6], Nina la Cour Freiesleben[3,6], Karina Banasik[1,3], Søren Brunak[1*], Henriette Svarre Nielsen[3,6*]

Methods S1. Additional information on data preprocessing and modeling

Fig. S1: Number of markers remaining after pruning by the Hobohm II algorithm according to eight cut-off levels for the Spearman correlation coefficient.

Fig. S2: Correlation between MSD markers

Fig. S3: PCA plots for log2-transformed signal values colored by plate for the six MSD panels.

Table S1: Outcome descriptions, definitions, adjustments, and exclusion criteria.

Table S2: Hyperparameter values

Table S3: Comparison of PREGCO and the Danish Medical Birth Registry (DMBR).

Table S4: Variables and sources

Table S5: Results from cross-validation of machine learning models.

Table S6: Birth weight-gestational duration ratio parameters estimated from the Danish Medical Birth Registry (DMBR).

Methods S1: Additional information on data preprocessing and modeling

Meso Scale Diagnostics data normalization

We measured 47 inflammatory markers using the V-PLEX Human Biomarker 54-Plex Kit from Meso Scale Diagnostics (MSD). Due to low-quality assay validation, we excluded all measurements from the Th17 panel, resulting in 47 assays on six panels to be included in the analysis.

To address the batch effect exerted by the individual 96-well plate setup, we explored the corrective effects of three data pre-processing methods and four normalization methods with the aim of removing batch effects at panel level.

The MSD V-PLEX kit is based on electrochemiluminescence technology, where light emission from SULFO-TAG labels is measured as light intensity ("signal"). The signal values are within the dynamic range of the assay linearly associated with the concentration of the measured target. While this principle is used for concentration determination by a measured standard curve, we here used the signals directly to avoid any noise introduced by the measurement of a standard curve.

Due to the multiplex setup, plate-based batch effects were assessed by panel, as technical variation was assumed to be equal across the four to ten assays measured per panel. Our first aim was to remove any observed variation based on the 16 plates the samples were run on. We compared the combination of three pre-processing methods: *a*) log2-transformation, *b*) method *a* plus removal of outliers based on principal component analysis (PCA)[1], and *c*) method *b* plus ComBat batch correction[2], and four normalization methods: *i*) no normalization, *ii*) median normalization[3], *iii*) quantile normalization[4], and *iv)* MA normalization[5]. To assess the effectiveness of normalization, we performed a visual inspection of PCA plots, density plots, and box plots.

Based on visual inspection of the PCA plots of the log2-transformed data, we defined individual outlier limits for the six panels removing samples that exceeded these limits. Consequently, we removed 17 outliers on Angio1, 13 outliers on Chem1, 11 outliers on Cyto1, 41 outliers on Cyto2, 8 outliers on Pro1, and 9 outliers on Vascu2 (see limits on Additional file 1: Fig. S3).

We found median normalization to sufficiently remove all batch effects observed at PC1 and PC2 and yielded overlapping curves with normal or near-normal distributions for the individual assays observed on density and box plots. Similar results were found for MA normalization, while the other methods yielded varying poorer results.

Similarity reduction of markers using Hobohm II

Hierarchical clustering showed high similarity between markers, indicating redundancy in the data set. To address this, we employed the Hobolm II algorithm as outlined in Hobohm *et al*, 1992[6]. We used Spearman correlation as the correlation metric and tested cut-off levels at 0.3, 0.4, 0.5, 0.6, 0.7, 0.8,

0.9, and 1 (Additional file 1: Fig. S1). We chose a cut-off of 0.5, resulting in 41 markers. Using this cut-off, we did not observe issues of collinearity in any of the statistical models.

Bayesian regression models

For all statistical analyses we employed Bayesian regression models. All models were fit using rstanarm or brms[7–9]. Unless otherwise specified, all models were run for 10,000 iterations (5,000 warm-up and 5,000 sampling) with default settings for the sampler. Convergence was assessed by calculating r-hat statistics, looking for divergences and making sure the sample did not exceed the maximum tree depth after warm-up. A model had converged if and only if (1) all r-hat values < 1.01, (2) no divergences, and (3) no iterations exceeding the maximum tree depth. All parameters were centered prior to model fitting and MSD inflammatory markers were standardized so that the interpretation follows changes in standard deviations. Furthermore, we also inspected correlations of the posterior distributions to identify issues of collinearity.

Bayesian robust linear regression

The Bayesian robust linear regression extends the classical ordinary least squares by assuming a Student t-distribution, thereby accommodating outliers. The degree of freedom is estimated directly from the data. Formally,

$$y \sim t(\nu,\ \mu,\ \sigma)$$

in which μ is the sum over the intercept and covariates,

$$\mu\ = b_0 +\ \sum_k \beta_k x_k$$

To complete the model, we specify a set of priors across the parameters in the model,

$$\beta_0 \sim N(0,\ 2.5)$$

$$\beta_k \sim t(7,\ 0,\ 0.5)$$

$$\sigma \sim N(0,\ 1)$$

$$\nu \sim N(0,\ 1)$$

Posterior model checking was done by simulating 100 draws from the posterior and comparing with the observed distribution.

Cause-specific parametric proportional hazards models

For analyzing the duration, or time to failure, we employed cause-specific parametric proportional hazards survival models, as implemented in rstanarm[8]. This takes into account that some women were lost to follow-up and are thus censored, and that there may be competing outcomes (e.g., induced labor, acute cesarean section, or scheduled cesarean section is a competing outcome to

spontaneous vaginal birth). We compared two baseline hazards (cubic B-spline and M-spline) for each outcome and visually inspected the estimated baseline hazard curve, versus the observed curve. Model priors follow default settings, except the regression coefficients (β_k) which were assigned a more conservative prior,

$$\beta_k \sim t(7,\ 0,\ 0.5)$$

The prior induces regularization by forcing coefficients towards zero, i.e., a null effect.

Birthweight to gestational age ratio

For the birth weight to gestational age ratio, the values were standardized according to the mean and standard deviation estimated from the Danish Medical Birth Registry (DMBR), including all births in 2020 (Additional file 1: Table S6). From the posterior, we defined a transformation,

$$P(X \leq x) = F(X|\mu, \sigma, \nu) - 0.5$$

in which X is the estimated value of the marker, and F is the Student-t cumulative density distribution parametrized by the mean, standard deviation, and degrees of freedom estimated from the DMBR. The parameters were estimated using a Bayesian robust linear regression. The resulting value represents the change from the 50th percentile for a one-unit change in the variable.

References

1. Merino GA, Fresno C, Netto F, Netto ED, Pratto L, Fernández EA. The impact of quality control in RNA-seq experiments. J Phys Conf Ser. 2016;705:012003.
2. Johnson WE, Li C, Rabinovic A. Adjusting batch effects in microarray expression data using empirical Bayes methods. Biostatistics. 2007;8:118–27.
3. Data normalization and standardization. Olink. https://olink.com/application/data-normalization-and-standardization/. Accessed 29 Dec 2022.
4. Bolstad BM, Irizarry RA, Åstrand M, Speed TP. A comparison of normalization methods for high density oligonucleotide array data based on variance and bias. Bioinformatics. 2003;19:185–93.
5. Hong M-G, Lee W, Nilsson P, Pawitan Y, Schwenk JM. Multidimensional Normalization to Minimize Plate Effects of Suspension Bead Array Data. J Proteome Res. 2016;15:3473–80.
6. Hobohm U, Scharf M, Schneider R, Sander C. Selection of representative protein data sets. Protein Sci. 1992;1:409–17.
7. Bürkner P-C. brms: An R Package for Bayesian Multilevel Models Using Stan. J Stat Softw. 2017;80:1–28.
8. Brilleman SL, Elci EM, Novik JB, Wolfe R. Bayesian Survival Analysis Using the rstanarm R Package. 2020; arXiv:2002.09633.
9. Goodrich B, Gabry J, Ali I, Brilleman S. rstanarm: Bayesian applied regression modeling via Stan. 2020.

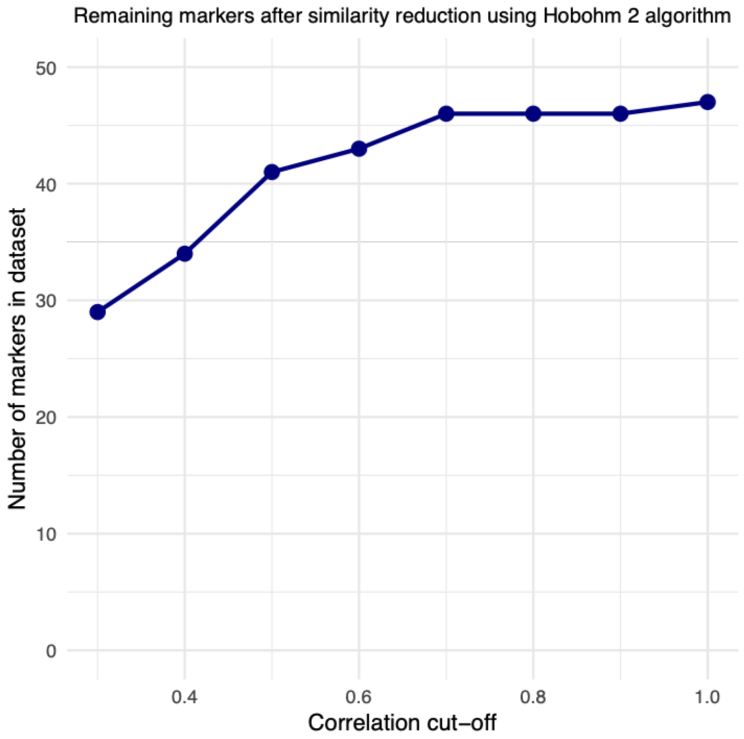

Fig. S1: Number of markers remaining after pruning by the Hobohm II algorithm according to eight cut-off levels for the Spearman correlation coefficient.

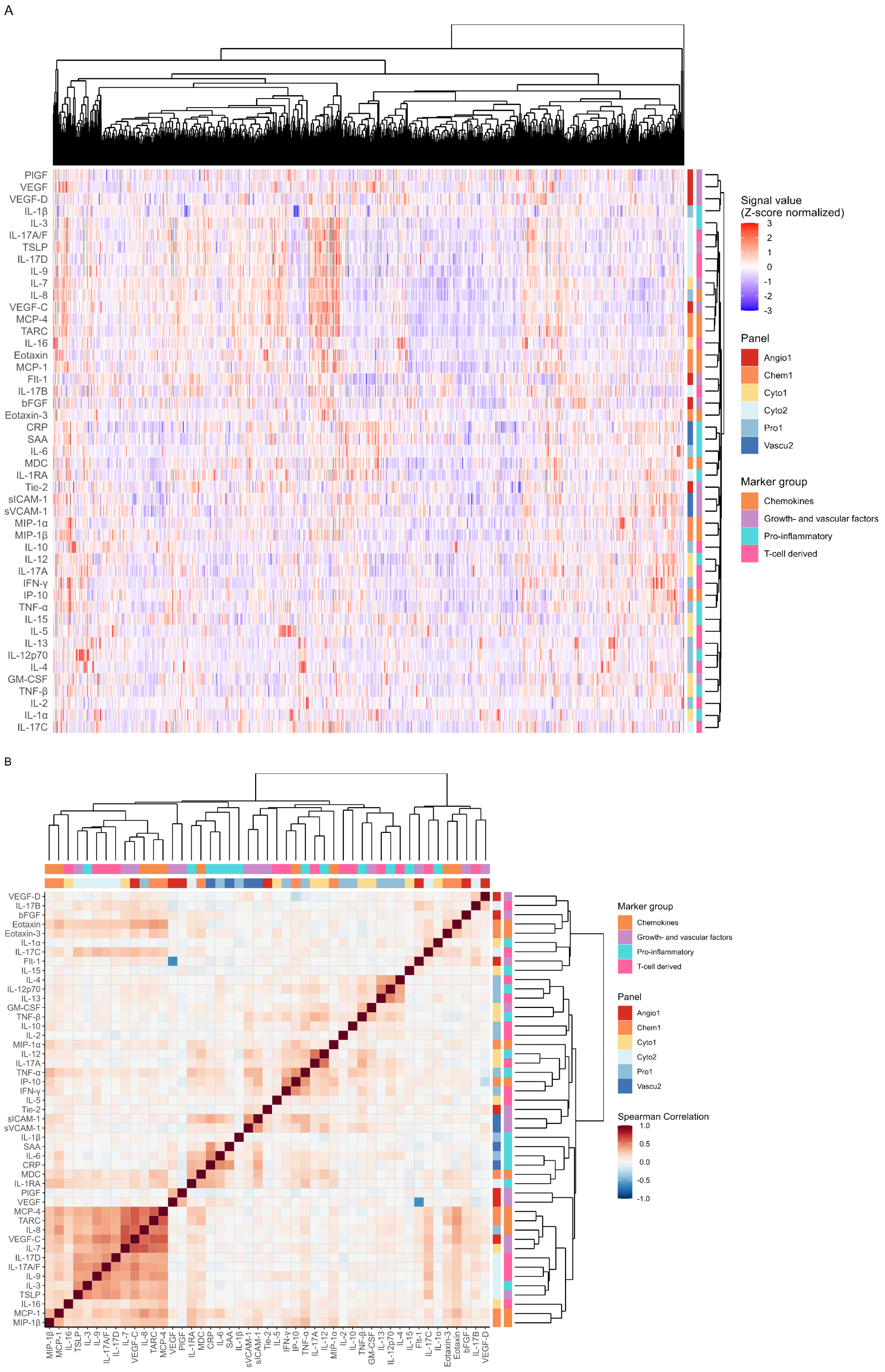

Fig. S2: Correlation between MSD markers. (a) Heatmap of women x markers, (b) Heatmap of markers x markers.

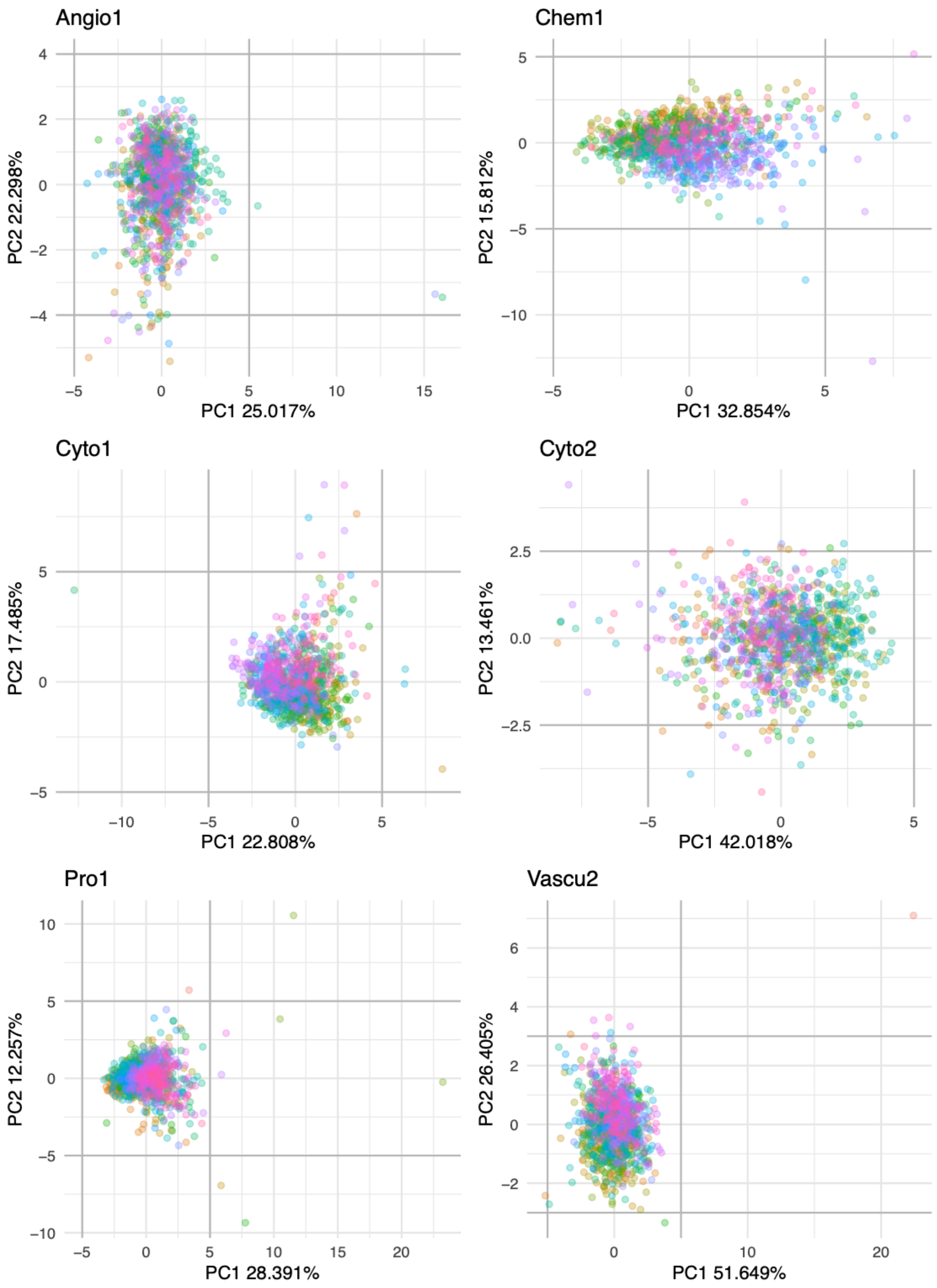

Fig. S3: PCA plots for log2-transformed signal values colored by plate for the six MSD panels. Grey lines indicate selected limits for outlier detection.

Table S1: Outcome descriptions, definitions, adjustments, and exclusion criteria.

Outcome	Definition	Outcome-specific adjustments	Exclusion criteria
Pre-eclampsia	Blood Pressure > 140 mmhg systolic and/or 90 mmHg diastolic & proteinuria	Previous number of live births,<br>Smoking,<br>PAPP-A MoM,<br>βHCG MoM,<br>ART (divided into IUI or IVF),<br>Ethnicity	Pre-gestational hypertension<br>Gestational hypertension diagnosed before inclusion<br>pre-eclampsia in previous pregnancy
Spontaneous vaginal birth	Birth not assisted by cesarean section or induction	Smoking,<br>Hemorrhage in early pregnancy,<br>UTI in current pregnancy,<br>Conisatio	n.a.
Gestational age at birth	Gestational age calculated from ultrasound.	Smoking,<br>Hemorrhage in early pregnancy,<br>UTI in current pregnancy,<br>Conisatio	n.a.
Preterm birth	Gestational age < 37+0	Smoking,<br>Hemorrhage in early pregnancy,<br>UTI in current pregnancy,<br>Conisatio	
Gestational diabetes mellitus (GDM)	Based on a two-hour 75 gram oral glucose tolerance test (OGTT); GDM with glucose ≥ 9.0 mmol/l in capillary whole blood or venous plasma	PCOS,<br>GDM in prior pregnancy	GDM diagnosed before inclusion<br>Diabetes Mellitus
Birth weight / gestational age ratio	Birth weight recorded immediately after birth by midwife or doctor.		
Severe Postpartum hemorrhage	1000 mL blood loss or more within 24 hours	ART (divided into IUI or IVF),<br>Hemorrhage in early pregnancy,<br>Cesarean section in prior pregnancy	
Acute cesarean section	Delivery by acute cesarean section,	Cesarean section in prior pregnancy	

	any degree		
Any complication	Pre-eclampsia, GDM, Severe PPH, Preterm birth, or acute cesarean section.	Previous number of live births,<br>Smoking,<br>PAPP-A MoM,<br>βHCG MoM,<br>ART (divided into IUI or IVF),<br>PE in prior pregnancy,<br>GDM in prior pregnancy,<br>PCOS,<br>UTI in current pregnancy,<br>Cesarean section in prior pregnancy,<br>Conisatio,<br>Ethnicity	Pre-gestational hypertension<br>Gestational hypertension diagnosed before inclusion<br>pre-eclampsia in previous pregnancy<br>GDM diagnosed before inclusion<br>Diabetes Mellitus (type 1 or 2)

Abbreviations: ART, assisted reproductive technology; GDM, gestational diabetes mellitus; IUI, intrauterine insemination; IVF, in vitro fertilization; MoM, multiple of the median; n.a., not applicable; OGTT, oral glucose tolerance test; PCOS, polycystic ovary syndrome; PE, Pre-eclampsia; PPH, postpartum hemorrhage; UTI, urinary tract infection

Table S2: Hyperparameter values

Model	Definition	Outcome-specific adjustments
Logistic regression	L1 penalty	1e-8 … 1 (log spaced)
LightGBM	Number of estimator	1 … 200
	Learning rate	0.0001 … 1 (log spaced)
	Max depth	3 … 30
	Subsample, individuals	0.2 … 0.8
	Subsample, feature	0.2 … 0.8
	Mininum child samples	50 … 200
	Maximum tree leaves	6 … 50
	L1 penalty	[0, 0.01, 1, 2, 5, 7, 10, 50, 100]
	L2 penalty	[0, 0.01, 1, 2, 5, 7, 10, 50, 100]

Table S3: Comparison of PREGCO and the Danish Medical Birth Registry (DMBR).

**Variable**		**PREGCO (n=1,049)**	**DMBR 2020 (n=60,573)**	**Difference**
Maternal age, years*		31.7 (4.5)	30.7 (4.7)	1.0 (0.72; 1.27, p < 0.001)
Pre-pregnancy BMI, m/kg^2*		24.1 (4.8)	24.8 (5.4)	-0.7 (-0.99; -0.41, p < 0.001)
Smoking during pregnancy**		40 (3.8%)	4,660 (7.7%)	0.49 (0.35; 0.67, p < 0.001)
Parity**	0	576 (54.9%)	22,644 (37.3%)	p < 0.001
	1	375 (35.7%)	20,421 (33.7%)	
	2	81 (7.7%)	10,011 (16.5%)	
	3+	17 (1.6%)	7,507 (12.4%)	
Number of Pregnancy Losses**	0	810 (77.2%)	52,145 (86%)	p < 0.001
	1	194 (18.5%)	6,965 (11.5%)	
	2	29 (2.8%)	1,230 (2.0%)	
	3+	16 (1.5%)	282 (0.5%)	
Sex of child, male**		521 (50.5%)	30,983 (51%)	0.96 (0.86; 1.07, p=0.44)

* Z-test; **χ^2 test

Table S4: Variables and sources

Group	Name	Timepoint measured / recorded and Source
Maternal Characteristics	Maternal age	Pre-pregnancy, EHR
	BMI	Pre-pregnancy, EHR
	Smoking	At inclusion, EHR
	Ethnicity	Pre-pregnancy, EHR
Current Pregnancy	ART	Pre-pregnancy, EHR
	Gestational age at inclusion, based on ultrasound	At inclusion, EHR
	GDM in current pregnancy	At inclusion, EHR
	COVID-19 antibodies	8-12th week scan and at inclusion, EHR
	Early hemorrhage	At inclusion, EHR
Fetal characteristics	Sex of child	At inclusion, EHR
	PAPP-A, MoM	8-12th week scan, EHR
	βHCG, MoM	8-12th week scan, EHR
	Gestational age, based on ultrasound	8-12th week scan, EHR
	Abdominal circumference	At inclusion, EHR
	Head circumference	At inclusion, EHR
	Femur length	At inclusion, EHR
Prior pregnancies	GDM in prior pregnancy	At inclusion, Questionnaire
	Pre-eclampsia in prior pregnancy	At inclusion, Questionnaire
	PPH in prior pregnancy	At inclusion, Questionnaire
	Preterm labor in prior pregnancy	At inclusion, Questionnaire
	Previous number of cesarean sections	At inclusion, Questionnaire
	Previous number of live births	At inclusion, Questionnaire
	Previous number of pregnancy losses	At inclusion, Questionnaire
Prior diseases	Conisatio	At inclusion, Questionnaire
	Endometriosis	At inclusion, Questionnaire
	PCOS	At inclusion, Questionnaire

Abbreviations: ART, assisted reproductive technology; BMI, body mass index; EHR, Electronic Health record; GDM, gestational diabetes mellitus; MoM, multiples of medians; PCOS, polycystic ovary syndrome

Table S5: Results from cross-validation of machine learning models. The highest ranking model for each outcome is highlighted in bold, based on the binary cross entropy from the inner CV.

		Development (inner CV)			Interval validation (outer CV)	
**Outcome**	**Model**	Binary Cross Entropy	ROC-AUC	AUPRC	ROC-AUC	AUPRC
Acute Sectio	**LASSO**	**0.216**	**0.638**	**0.130**	**0.590 (0.515-0.667)**	**0.086 (0.057-0.126)**
	LightGBM	0.216				
Any complication	**LASSO**	**0.599**	**0.630**	**0.457**	**0.615 (0.576-0.655)**	**0.421 (0.367-0.477)**
	LightGBM	0.602				
Gestational Diabetes	LASSO	0.268				
	**LightGBM**	**0.266**	**0.697**	**0.219**	**0.708 (0.644-0.766)**	**0.176 (0.126-0.238)**
Pre-eclampsia	LASSO	0.150				
	**LightGBM**	**0.149**	**0.655**	**0.125**	**0.672 (0.580-0.758)**	**0.073 (0.042-0.115)**
Preterm birth	LASSO	0.172				
	**LightGBM**	**0.171**	**0.610**	**0.118**	**0.564 (0.460-0.66)**	**0.071 (0.041-0.132)**
Severe PPH	LASSO	0.349				
	**LightGBM**	**0.349**	**0.628**	**0.198**	**0.587 (0.530-0.638)**	**0.152 (0.117-0.193)**

Table S6: Birth weight-gestational duration ratio parameters estimated from the Danish Medical Birth Registry (DMBR).

Parameter	Median	95% bCI
μ	12.61	12.59-12.62
σ	1.54	1.53-1.56
ν	6.88	6.54-7.24
